# Validation of a therapeutic range for nitisinone in patients treated for tyrosinemia type 1 based on reduction of succinylacetone excretion

**DOI:** 10.1002/jmd2.12023

**Published:** 2019-03-14

**Authors:** Rhona M. Jack, C. Ronald Scott

**Affiliations:** ^1^ Department of Laboratories Seattle Children's Hospital Seattle Washington; ^2^ Department of Laboratory Medicine University of Washington Seattle Washington; ^3^ Division of Genetic Medicine, Department of Pediatrics University of Washington School of Medicine Seattle Washington

**Keywords:** nitisinone, NTBC, Orfadin, succinylacetone, tyrosinemia

## Abstract

The drug nitisinone (NTBC; Orfadin, Vienna, Austria) has been used for the treatment of hereditary tyrosinemia type‐1 since 1991. Nitisinone effectively blocks the metabolism of tyrosine to prevent the formation of the toxic compound succinylacetone (and precursor fumarylacetoacetate) in affected children. Monitoring of plasma drug levels and urine succinylacetone can be used to assess compliance and adequate dose of drug. We present retrospective data from patient monitoring for over 10 years that provide validation of a target therapeutic range for nitisinone of 40 to 60 μmol/L. The target nitisinone range is justified as valid based on reduction of succinylacetone excretion. There was no statistical significance in succinylacetone excretion in mmol/mol creatinine above a level of 40 μmol/L plasma NTBC (*P* > 0.05).

**Abbreviations**
FA: formic acid
FAH: fumarylacetoacetate hydrolase
FDA: Food and Drug Administration
GCMS: gas chromatography/mass spectrometry
HT‐1: hereditary tyrosinemia type 1
IS: internal standard
LC‐MS/MS: liquid chromatography‐tandem mass spectrometry
NTBC: 2‐[2‐nitro‐4‐trifluoromethylbenzoyl]‐cyclohexane‐1,3‐dione
SUAC: succinylacetone
TDM: therapeutic drug monitoring

## 1 INTRODUCTION

**Figure 1:**
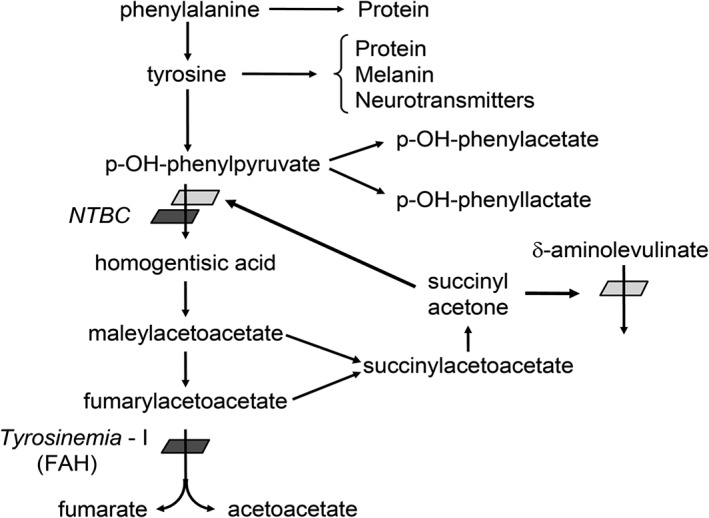
Metabolism of tyrosine and block in HT‐1. The block in the enzyme fumarylacetoacetate hydrolase (FAH) causes buildup of fumarylacetoacetate and succinylacetone, which causes hepatic and renal toxicity, as well as inhibition of porphobilinogen synthase activity, resulting in accumulation of δ‐aminolevulinic acid. NTBC inhibits the enzyme 4‐hydroxyphenylpyruvate dioxygenase, prior to the accumulation of toxic compounds

The drug nitisinone (2‐(2‐nitro‐4‐trifluoromethylbenzoyl)‐cyclohexane‐1,3‐dione; NTBC) was initially approved by the FDA as Orfadin (Orphan Pharmaceuticals) in January 2002 for use in Hereditary Tyrosinemia type 1 (HT‐1) (Prescribing information, Orphan Pharmaceuticals). Prior to FDA approval, Orfadin was listed as an orphan drug in 1995 by the Office for Orphan Product Development. NTBC was originally considered for use as an herbicide due to its inhibition of 4‐hydroxyphenylpyruvate dioxygenase.^1^ The realization that NTBC blocked the proximal tyrosine pathway led to investigation regarding the use of the compound in HT‐1 by blocking the proximal tyrosine pathway and preventing the formation of fumarylacetoacetate and succinylacetone (Figure 1).

Tyrosinemia type 1 is an autosomal recessive condition caused by a deficiency of fumarylacetoacetate hydrolase (FAH). Symptoms typically develop in early infancy (<6 months of age) or before 2 years of life.^2^, ^3^ Untreated children develop acute liver failure and renal dysfunction, leading to rickets and growth failure. Patients carry a high risk of hepatocellular carcinoma, which is often the cause of death in an untreated individual. Liver transplant has been used as treatment, although it is not curative. Lindstedt et al.^4^ first reported the treatment for hereditary tyrosinemia with NTBC in 1992 and demonstrated the decrease in urine and plasma succinylacetone in response to daily oral NTBC. The first clinical trial using NTBC for HT‐1 began in 1991,^5^ which documented significant improvement in the clinical course of the disease. Biochemical markers for HT‐1 include elevated plasma tyrosine (as well as phenylalanine and methionine), elevated plasma and urine succinylacetone, delta‐aminolevulinic acid, and alpha‐fetoprotein.

In the United States, most states include detection of plasma succinylacetone as target for HT‐1 in their newborn screening algorithms. Treatment with NTBC is most effective if patients are diagnosed early, making this an ideal disease for newborn screening. When children are detected by newborn screening and treatment (both low protein diet and NTBC) initiated early, patients show excellent clinical outcome without liver or renal disease.^6^, ^7^ If treatment is delayed, renal and hepatic damage may progress to liver cirrhosis.^8^

The blood concentration of NTBC generally recommended is 40 to 60 μmol/L.^2^ We looked retrospectively at 144 paired plasma NTBC levels and urine succinylacetone levels in 34 patients over a 10 year period of time, to validate that the recommended plasma drug concentration was indeed reducing the succinylacetone as desired.

## 2 METHODS

### 2.1 Measurement of succinylacetone by GC/MS

Succinylacetone was measured by selected ion monitoring gas chromatography/mass spectrometry (GC/MS) in splitless mode. One milliliter of urine was acidified, extracted with ethylacetate and ether, dried down, and derivatized with BSTFA.^9^ A stable isotope internal standard (^13^C_5_‐succinylacetone), obtained from Cambridge Isotope Laboratory was included in the extraction, and used for quantitation. Target ion for quantitation was 212 m/z for succinylacetone, and 217 m/z for the internal standard. A qualifier ion was used for peak identification confirmation; 227 m/z for succinylacetone and 232 for internal standard. The assay has an analytical measurement range of 0.2 to 40 μmol/L, with dilution above 40 μmol/L to 400 μmol/L.

### 2.2 Measurement of NTBC by LC‐MS/MS

NTBC was measured by liquid chromatography/tandem mass spectrometry (LC‐MS/MS).^10^, ^11^ A Waters QuattroMicro mass spectrometer coupled to an Agilent 1100 HPLC or XEVO TQMS tandem mass spectrometer coupled to a Waters Acquity system was used for analysis. Twenty microliters of plasma sample was acidified with 0.2% formic acid (FA) in water, and then treated with acidified acetonitrile (0.2% FA) containing mesotrione as the internal standard. The plasma proteins were precipitated and the samples were centrifuged. The supernatant was then injected into an LC‐MS/MS system in positive electrospray ionization mode. NTBC and mesotrione were separated by an acetonitrile/water (0.1%FA) gradient using a Waters SymmetryShield RP18 column, 3.5 μm particle size, and 2.1 × 30 mm column size or a Waters Acquity UPLC BEH C18 1.7 μm, 2.1 × 30 mm column. Analytes were quantified by appropriate multiple reaction monitoring transitions; 320.1 > 218.0 for NTBC, 340.1 > 228.0 for mesotrione IS.

## 3 RESULTS

Data were segregated by NTBC level as follows: <20, 20‐30, 30‐40, 40‐50, 50‐60, and > 60 μmol/L. In reviewing all the data, mean succinylacetone excretion was 0.61 μmol/L (SD 1.2) and 0.15 mmol/mol creatinine (SD 0.32). Mean NTBC was 47.0 μmol/L (SD 16.7). Average succinylacetone excretion decreased at increasing NTBC plasma levels as follows:

**Table:** 

Plasma NTBC μmol/L	Average urine SUAC μmol/L	Average urine SUAC mmol/mole creatinine	Number of sample pairs
0‐20	3.89	0.88	7
20‐30	0.8	0.23	17
30‐40	0.45	0.17	29
40‐50	0.43	0.08	33
50‐60	0.35	0.08	30
>60	0.43	0.06	28
		Total	144

**Figure 2:**
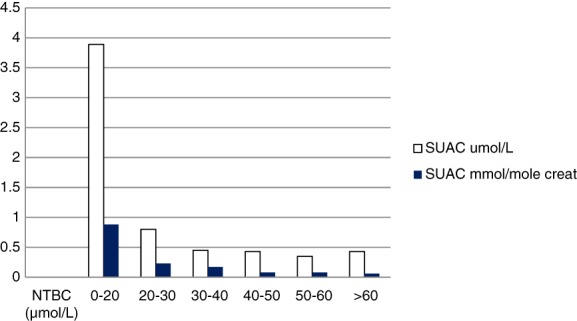
Succinylacetone excretion at increasing plasma NTBC levels. As NTBC blood levels increase, there is no significant difference in the mmol SUAC/mol creatinine excreted above an NTBC plasma level of 40 μmol/L (*P* > 0.05), supporting a therapeutic range of 40 to 60 μmol/L. There is significant difference in succinylacetone excretion below 40 μmol/L (*P* < 0.05)

There was no statistical significance in excretion of succinylacetone in μmol/L above a level of 30 μmol/L plasma NTBC (*P* > 0.05; paired *t* test for a comparison of two means, Graph Pad, 2018). There was no statistical significance in excretion in mmol/mol creatinine above a level of 40 μmol/L plasma NTBC (*P* > 0.05) (Figure 2). Since accuracy of quantitation in urine is improved when normalized to creatinine, we feel this confirms the recommendation that if therapeutic drug monitoring (TDM) is performed, a target NTBC level between 40 and 60 μmol/L is optimal. It was also noted that among patients with excretion >0.25 mmol/mol creatinine, 83% had NTBC levels less than the mean of 47.0 μmol/L.

In our laboratory, we have set an upper limit of desirable succinylacetone excretion for patients on NTBC as 1.5 μmol/L and 0.25 mmol/mole creatinine. These numbers were justified by our observation that in 99% of treated patients with NTBC levels over 40 μmol/L, succinylacetone excretion was <1.5 μmol/L and < 0.25 mmol/mole creatinine. In patients with NTBC levels <40 μmol/L, 19% had succinylacetone excretion >1.5 μmol/L, and 28% had excretion >0.25 mmol/mole creatinine.

## 4 DISCUSSION

Treatment for HT‐1 is based on dietary restriction of protein and drug therapy. Since treatment with NTBC blocks the tyrosine pathway, it is logical that a side effect of treatment would be an elevated tyrosine. For this reason, dietary protein restriction with minimization of tyrosine and phenylalanine is a primary nutritional goal. Maintenance of plasma tyrosine at a level of 200 to 600 μmol/L is recommended.^2^

The dose of NTBC recommended is 0.5 mg/kg orally (maximum 1 mg/kg), twice daily. Dose is titrated based on biochemical and or clinical response (Orfadin prescribing information). Although low in toxicity, the most common adverse reactions are elevated tyrosine (>10%), thrombocytopenia, leukopenia, conjunctivitis, corneal opacity, keratitis, photophobia, eye pain, cataracts, granulocytopenia, rash, and alopecia (all <3%) (Orfadin prescribing information). The half‐life is 54 hours. Although succinylacetone can be measured as a marker for biochemical response to adequate NTBC dose, NTBC can also be measured for TDM to ensure compliance and adequate dose, or to assess toxicity if suspected. We have no correlating NTBC dosage or time of blood draw information, dietary information, or blood tyrosine levels in this data set. A more complete prospective study would include this information. The intention of this publication is only to compare urine succinylacetone excretion with concurrent NTBC blood levels, and provide some support for a practice already in place. Laboratory instructions to providers, however, recommend blood collection for NTBC levels 30 minutes prior to the next dose. This is standard for monitoring of trough drug levels. We assume most of the patient drug levels are measured at steady state.

Our laboratory has routinely monitored both urine succinylacetone excretion and plasma NTBC levels in patients on treatment for HT‐1. Treatment should lead to normalized porphyrin metabolism, minimal excretion of succinylacetone, and undetectable plasma succinylacetone, although it may take up to 3 months before the level of plasma succinylacetone is normalized after the start of NTBC treatment. An argument can be made for measurement of plasma succinylacetone as a better measure for monitoring endogenous succinylacetone. It is true that measurement in plasma is likely to be more reproducible, as plasma measurement avoids variation in extraction due to urine concentration differences, and the need to relate excretion to creatinine concentration. In this retrospective data set, only urine succinylacetone and plasma NTBC were requested and measured. Our conclusions correlate well, however, with those of Kienstra et al.,^12^ who determined that succinylacetone was only detectable in blood spots as measured by tandem MS/MS in patients with NTBC blood levels <44.3 μmol/L.

Since 99% of treated patients monitored at our institution who had NTBC levels over 40 μmol/L also had succinylacetone excretion <1.5 μmol/L and < 0.25 mmol/mole creatinine, and there was no statistical significance in the excretion level in patients with NTBC above 40 μmol/L, we feel the target for blood levels of 40 to 60 μmol/L^2^ is supported. This is also consistent with the theoretical effectiveness of NTBC based on the published inhibitory constant of 5 nM,^13^ which predicts that a concentration of 35 μM NTBC will inhibit 99.9% of the activity of 4‐hydroxyphenylpyruvate dioxygenase.

## CONFLICTS OF INTEREST

The authors have no conflict of interest.

## AUTHOR CONTRIBUTIONS

Rhona Jack contributed to conception and design, data acquisition, statistical analysis, interpretation of data, writing, editing, and guarantor.

C. Ronald Scott contributed to conception, interpretation of data, critical editing, and review.
